# Automation of Rietveld refinement through machine learning

**DOI:** 10.1107/S1600576726001494

**Published:** 2026-03-26

**Authors:** Suk Jin Mun, Yoonsoo Nam, Sungkyun Choi

**Affiliations:** a Center for Integrated Nanostructure Physics Institute for Basic Science (IBS) Suwon 16419 Republic of Korea; b Sungkyunkwan University, Suwon 16419, Republic of Korea; c Rudolf Peierls Centre for Theoretical Physics, University of Oxford, Parks Road, Oxford OX1 3PU, United Kingdom; d Center for Van der Waals Quantum Solids Institute for Basic Science (IBS) Pohang 37673 Republic of Korea; DESY, Hamburg, Germany

**Keywords:** machine learning, deep learning, convolutional neural networks, automatic Rietveld refinements, powder X-ray diffraction

## Abstract

Rietveld refinement is a widely used technique for determining crystal structures from powder X-ray diffraction data. Despite its broad applicability, the refinement process often requires substantial manual effort and expert knowledge, which limits the pace of materials research. Here, we introduce a methodology based on convolutional neural networks that enables automated Rietveld refinement. A systematically generated training dataset, which incorporates diverse structural and profile parameters, allows the network to capture complex pattern–structure relationships effectively. A refined crystal structure is directly extracted from the experimental powder X-ray diffraction pattern in a single inference step. The approach is validated using benchmark datasets of CeO_2_, Tb_2_BaCoO_5_ and PbSO_4_, achieving reliability factors comparable to those obtained from conventional methods. This work establishes a generalizable methodology by providing valuable insights into the development of autonomous diffraction analysis, with the potential to accelerate materials discovery and characterization.

## Abstract 2

A machine-learning-driven methodology is introduced to automatically refine crystal structures using powder X-ray diffraction data.

## 1. Introduction

Throughout numerous scientific fields, including physics, chemistry, biology, metallurgy, pharmaceutics and mineralogy, as well as industries, researchers determine the structural parameters of crystalline materials from powder X-ray diffraction (XRD) data using Rietveld refinement (Rietveld, 1969 ▸). To achieve this, well-established software tools (Petříček *et al.*, 2014 ▸; Rodríguez-Carvajal, 1993 ▸; Toby & Von Dreele, 2013 ▸) are typically employed. However, this conventional approach relies heavily on manual iterative adjustments (McCusker *et al.*, 1999 ▸) guided by individual expertise. This method requires a significant investment of time and may occasionally result in the generation of physically unrealistic structures. Consequently, there has been considerable motivation within the community to automate this process.

Employing a machine learning (ML) methodology has become increasingly feasible due to substantial recent advancements (Jordan & Mitchell, 2015 ▸; Butler *et al.*, 2018 ▸). Deep learning, a subfield of ML, utilizes neural networks, such as convolutional neural networks (CNNs). They are trained on specific datasets for various tasks, such as image classification (He *et al.*, 2016 ▸) and natural language processing (Devlin *et al.*, 2019 ▸). Over the past decade, crystallography and structural science have increasingly incorporated deep learning algorithms to automatically extract various aspects of structural information from powder XRD data (Billinge & Proffen, 2024 ▸). This includes phase identification using artificially generated XRD patterns (Lee *et al.*, 2020 ▸), autonomous phase identification (Szymanski *et al.*, 2021 ▸, 2023 ▸), and determination of Bragg peak positions (Liu *et al.*, 2022 ▸), space groups (Park *et al.*, 2017 ▸) and unit cells using pair distribution functions (Guccione *et al.*, 2023 ▸). Also, a neural network for parameter quantification, named the Parameter Quantification Network (PQ-Net), provides quantitative analysis such as scale factors, crystallite sizes and lattice parameters, with potential as a method for real-time structural analysis (Dong *et al.*, 2021 ▸). However, most deep learning studies in this area focus on classifying the diffraction data by identifying phases in experimental powder XRD data.

On the other hand, studies that aim to automatically determine the crystal structure via Rietveld refinement remain few. For instance, the *PowderBot* program (Feng *et al.*, 2019 ▸) was developed for better decision making in Rietveld refinement on the basis of a reinforcement learning algorithm. Also, a blackbox optimization algorithm was introduced (Ozaki *et al.*, 2020 ▸). However, they are still based on the conventional trial-and-error processes, a concept of manual Rietveld refinement. Thus, the automation of Rietveld refinement to determine the crystal structure via deep learning has yet to be thoroughly explored.

Herein, we introduce the Rietveld Analysis Pipeline with Intelligent Deep Learning (RAPID). We automate Rietveld refinement using CNN training with simulated data, by minimizing manual intervention. We validate the model against experimental XRD data for CeO_2_, Tb_2_BaCoO_5_ and PbSO_4_. We achieve reliability factors comparable to those obtained through traditional Rietveld refinement methods. The automated refinement, utilizing ML, is further demonstrated by presenting physically meaningful correlation matrices. This approach provides a rapid and scalable solution for both fundamental research and industrial applications.

The paper is organized as follows. Section 2 presents the computational details. An overview of this study is described in Section 2.1, followed by explanations for the data generation in Section 2.2 and CNN architecture in Section 2.3. Section 3 addresses the experimental aspects. Our results are outlined in Section 4, followed by discussion in Section 5. The conclusions are summarized in Section 6. The appendices provide detailed computational information.

## 2. Computational details

### 2.1. Overview

**Figure 1:**
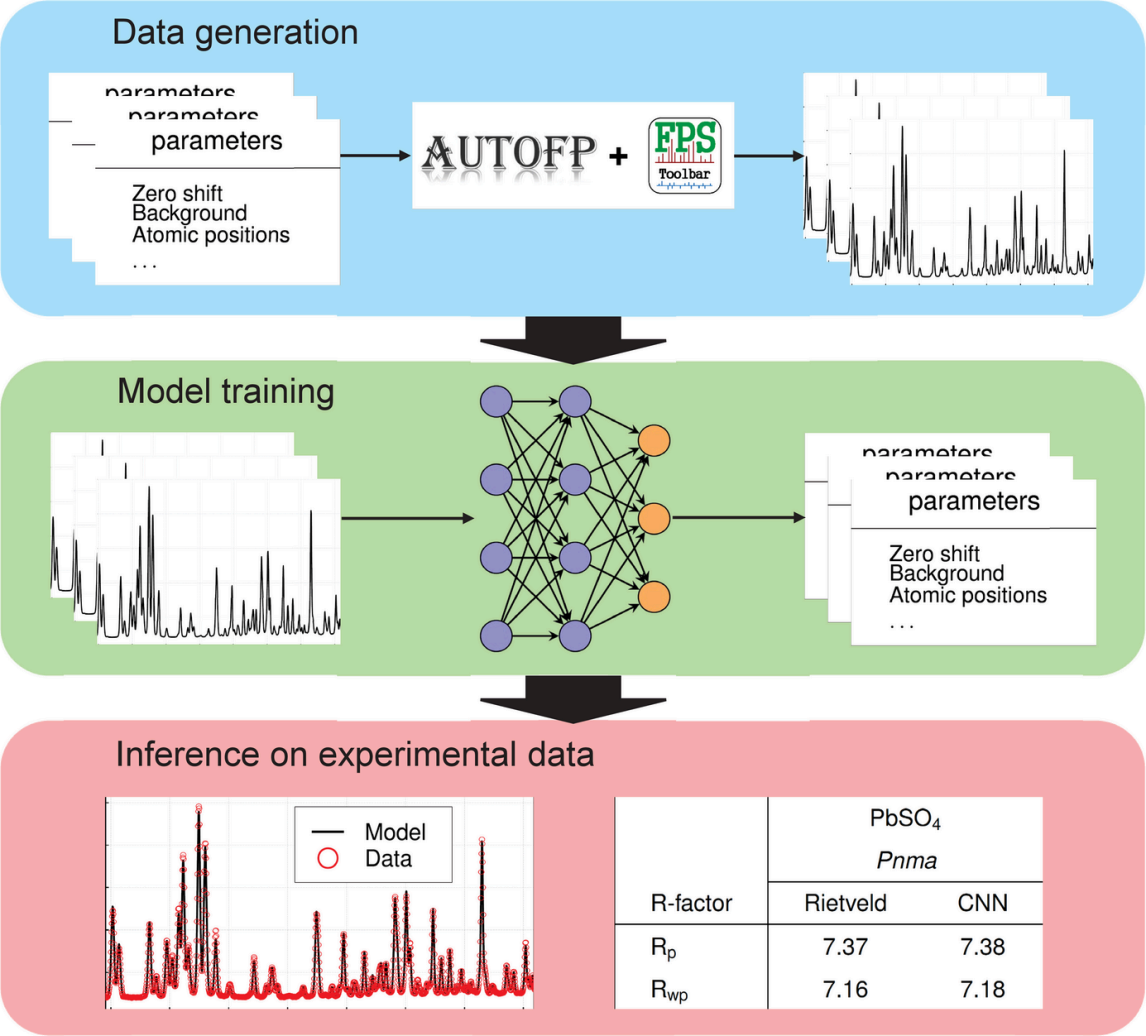
Summary of the workflow. (*a*) Data generation. (*b*) Model training. (*c*) Inference on experimental data.

Fig. 1 ▸ summarizes the workflow proposed in this study. We first generated a large library of physics-based simulated powder diffraction patterns [Fig. 1 ▸(*a*)]. A CNN was then trained to predict structural and profile parameters directly from these patterns [Fig. 1 ▸(*b*)]. When applied to experimental data, the trained CNN delivers parameter estimates in a single inference, achieving *R* factors that match those of conventional Rietveld refinement [Fig. 1 ▸(*c*)]. Details of the workstation and computational performance are included in Appendix *A*[App appa]).

### 2.2. Augmented data generation

In this study, we assume prior knowledge of the crystalline phase, which can be obtained through either conventional or deep learning approaches (Lee *et al.*, 2020 ▸; Szymanski *et al.*, 2021 ▸; Mikhalychev & Ulyanenkov, 2017 ▸) (see Appendix *B*[App appb]).

In the pipeline illustrated in Fig. 1 ▸(*a*), we first generated the training dataset, which comprises tuples of XRD patterns along with their corresponding profile and structural parameters. Using *AutoFP* (Cui *et al.*, 2015 ▸) and *FullProf* (Rodríguez-Carvajal, 1993 ▸), we automatically generated the XRD patterns from a crystallographic information file (CIF), which is a standardized text file (Hall *et al.*, 1991 ▸). This file contains essential structural information, including lattice parameters, space groups, atomic types, fractional atomic coordinates, occupancies and thermal parameters (*B*_iso_). The simulated XRD data include realistic profile parameters, such as zero shift, background coefficients, a scale factor and peak-shape coefficients (*U*, *V*, *W*). Additional parameters, such as asymmetry coefficients and scan ranges, were also incorporated as needed. The PCR file, an input for *FullProf*, contains all information related to the structural and profile parameters.

We subsequently generated augmented PCR files for CNN training. In deep learning, training samples are augmented to prevent overfitting by increasing data diversity, a strategy commonly employed for both image (Krizhevsky *et al.*, 2012 ▸) and scientific datasets (Kovács *et al.*, 2021 ▸). In accordance with this principle, we typically generated 10 000 PCR files per crystal structure, each incorporating systematically perturbed parameters to create unique parameter sets. The data augmentation employs a hybrid sampling strategy: 50% of the samples utilize Sobol sequences (Case *et al.*, 2016 ▸) for quasi-random uniform coverage of the parameter space, and the remaining 50% employ random sampling to ensure diversity. This approach guarantees a comprehensive exploration of combinations of physically plausible parameters while maintaining both systematic coverage and stochastic variation. To reduce computational complexity during training, we fixed the background coefficients during augmentation. This is also consistent with a normal refinement process to fix backgrounds to stabilize the structural determination (Kisi & Howard, 2008 ▸). In this way, we minimized unphysical correlations between the parameters (see Appendix *C*[App appc]).

After generating the XRD patterns, we implemented a quality control routine that classifies each pattern–information pair as Close, Boundary or Invalid according to the residual weighted profile *R*_wp_ or *R* factor – widely used indicators for fit reliability in Rietveld analysis (Martín-Rodríguez *et al.*, 2025 ▸). An adaptive feedback loop was subsequently employed to adjust the parameter distributions, ensuring that ∼80% of the data fall within the Close class and 20% within the Boundary class, while the Invalid class is discarded. Maintaining this ratio prevents the training set from being dominated by trivial or inferior examples. The physics-informed perturbations introduced during the simulation, combined with this quality-controlled sampling, expose the neural network to realistic laboratory conditions and effectively mitigate the scarcity of large experimental datasets available for training (see Appendix *D*[App appd] for the details).

### 2.3. CNN architecture and training

**Figure 2:**
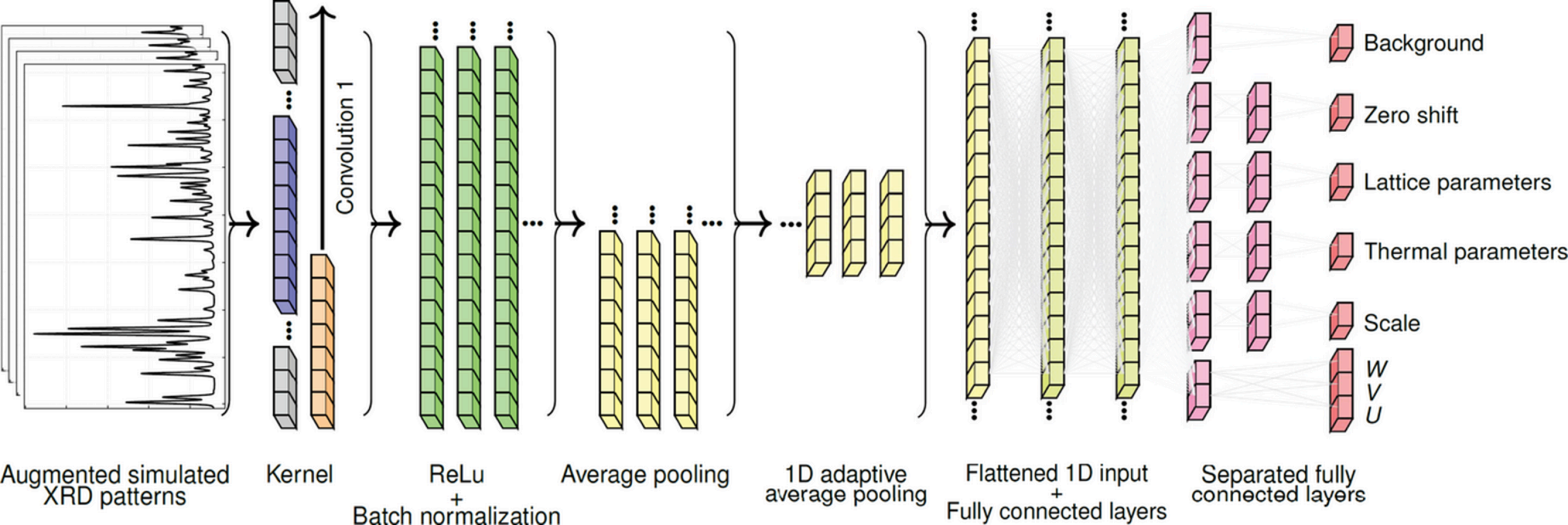
The CNN architecture. The diagram simplifies five convolutional layers when extracting features from the augmented simulated XRD spectra, via convolution, kernel operations, ReLU + BatchNorm and average pooling. The architecture includes one-dimensional adaptive average pooling to produce fixed-length features, followed by flattening to a one-dimensional input. The full model contains two shared fully connected layers with batch normalization, ReLU activation and dropout regularization, though these are not all shown for brevity. Following the shared layers, the architecture branches into separate unshared fully connected layers, where each parameter has its own dedicated fully connected layer.

Fig. 2 ▸ depicts the CNN architecture utilized in this study. The CNN is trained to predict structural and profile parameters from XRD patterns. It consists of five sequential convolutional blocks with decreasing kernel sizes, allowing for the extraction of features at varying scales. The initial input comprises a batch of XRD patterns with intensity values along the 2θ axis.

The first block describes a convolutional operation with a kernel size of 7 and a padding of 3 (convolution 1 in Fig. 2 ▸). This larger kernel effectively captures broad patterns in the diffraction data, transforming the single-channel input into 64 feature channels. After the convolution, the rectified linear unit (ReLU) activation function (Glorot *et al.*, 2011 ▸) and batch normalization (Ioffe & Szegedy, 2015 ▸) are applied, followed by average pooling (LeCun *et al.*, 1998*a* ▸) with a pool size of 2, which reduces the spatial dimensions by half. This sequence of operations is repeated up to the fifth convolutional layer, during which the spatial dimensions are consistently reduced. The multi-channel feature map is then flattened into a one-dimensional vector of length 512 and projected through fully connected layers with dropout regularization to create a robust feature representation. This shared representation is fed into fully connected layers that predict the normalized structural and profile parameters (Fig. 2 ▸). Unlike conventional CNN architectures that use a single shared output layer, our design employs separated fully connected layers for each parameter type. After the shared feature extraction layers, the network branches into independent pathways where each structural and profile parameter has its own dedicated fully connected layer that operates independently of the others. This departure from conventional architectures, while unconventional in CNN design, was adopted because it improved the generalization performance of the model compared with using traditional shared layers. Parameter normalization prevents the profile with the largest magnitude from dominating training and is reversed during analysis. Our CNN performs a regression task in contrast to more common classification tasks (see Appendix *E*[App appe] for the detailed algorithm and training optimization).

## 3. Experimental details

Experimental powder XRD data were used with the deep learning pipeline described in Section 2 for automatic refinements. The three experimental XRD datasets (CeO_2_, Tb_2_BaCoO_5_ and PbSO_4_) were obtained from *FullProf* examples (Rodríguez-Carvajal, 1993 ▸). For CeO_2_, the scan covered 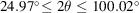 with a fixed 0.025° step. For Tb_2_BaCoO_5_, the range was 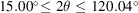 with a 0.020° step. For PbSO_4_, a long-range pattern was collected in the range 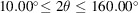 using a constant increment of 0.05°. No background subtraction or peak-profile smoothing was applied; therefore, the diffraction patterns were utilized in all subsequent analyses in their original instrument-recorded form.

## 4. Results

### 4.1. Automatic refinements by CNN

To evaluate the CNN results, we compared the *R* factors obtained from the CNN with those derived from traditional Rietveld refinement across three example crystal structures: CeO_2_, Tb_2_BaCoO_5_ and PbSO_4_. A conventional manual structural refinement was performed using *FullProf* to establish benchmark structural and profile parameters. The refinement included lattice constants, scale factors, isotropic displacement parameters, peak-shape coefficients (*U*, *V*, *W*) and asymmetric parameters, with the background fixed to a six-term polynomial.

**Figure 3:**
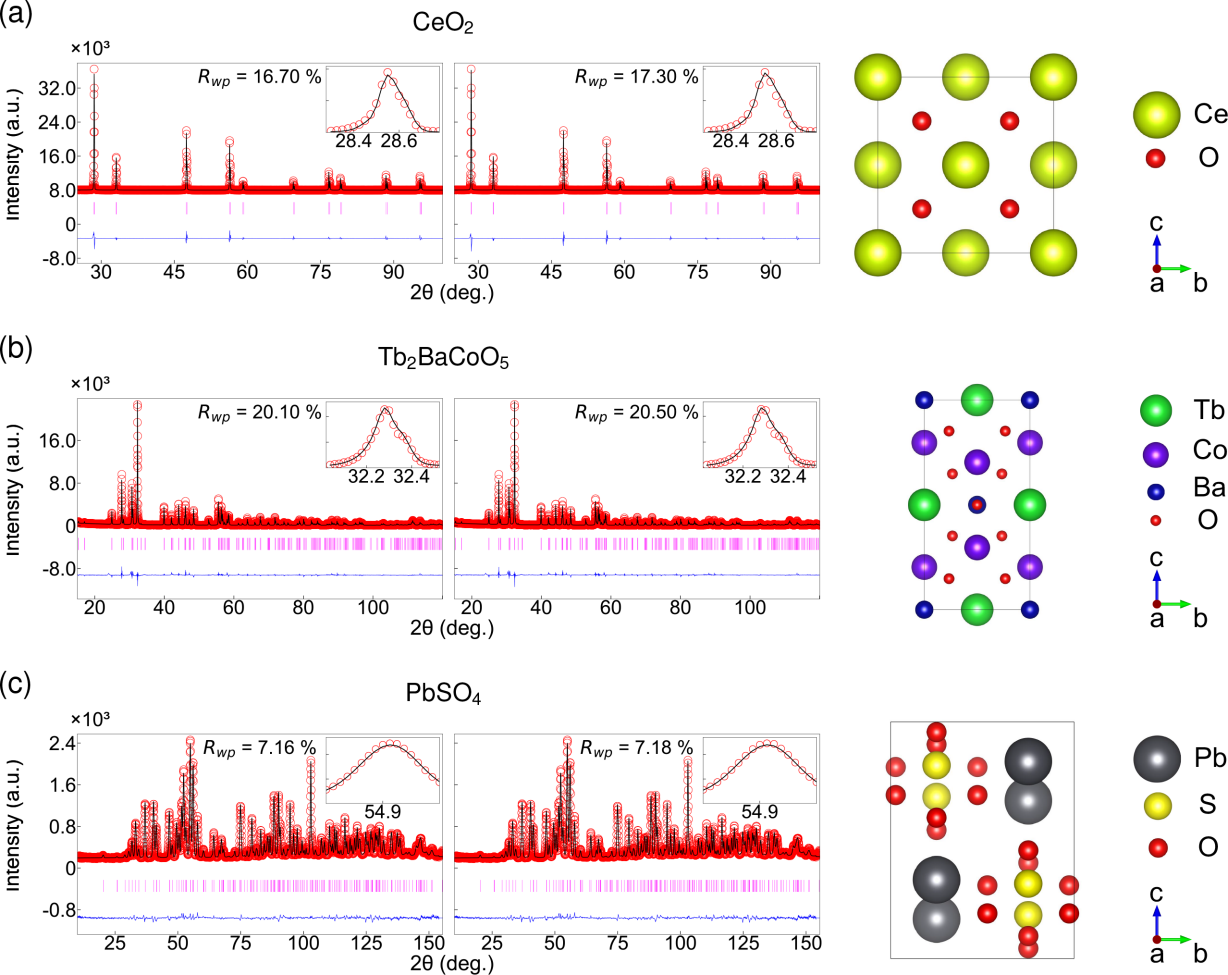
Rietveld refinement results for three representative crystal structures. Row (*a*) shows CeO_2_ with a fluorite-type cubic structure *Fm*
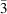
*m*, row (*b*) shows Tb_2_BaCoO_5_ with an orthorhombic structure *Immm* and row (*c*) shows PbSO_4_ with an orthorhombic structure. The left and middle panels display conventional and CNN ML refinements, respectively. Each refinement shows observed data as red circles, calculated patterns as black lines, difference curves in blue and Bragg positions as magenta markers. Insets provide zoomed views of the strongest peak. The *R*_wp_ values demonstrate that CNN-predicted parameters achieve refinement quality matching conventional approaches with differences of less than 1%. The right panels show the refined crystal structure obtained from the CNN method.

Fig. 3 ▸ compares the automatic and manual Rietveld refinement results for the three structural examples. It demonstrates that the Rietveld refinement initialized with CNN-predicted parameters achieves a fit quality and parameter values that are nearly identical to those obtained through conventional refinement. Table 1 ▸ summarizes structural and profile parameters extracted from the CNN and traditional Rietveld refinement, indicating the potential of the model to replace manual labor. All CNN-estimated parameters are comparable to the conventional Rietveld results within the uncertainty. The average difference in *R*_wp_ across the three samples is 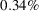, which is very good. All parameters were obtained in a single inference step via the trained CNN algorithm.

If we consider the refinement results case by case, those for CeO_2_ show excellent agreement between the predicted parameters and conventionally refined parameters, as illustrated in the first row of Fig. 3 ▸(*a*). *R*_wp_ converges to 17.3% with the CNN initial parameters, while it is 16.7% with manual refinement (see Appendix *F*[App appf] for representative *R* factors). This small difference of 0.6% highlights the CNN’s ability to provide refinement-ready starting parameters that are comparable to those optimized through manual methods. All refined structural parameters, including the lattice parameter *a* and *B*_iso_, are in agreement within their respective uncertainties. The values predicted by the CNN were very close to the final refined values before any additional least-squares refinement iterations. The slight adjustments made during the refinement were minimal, indicating that the network provides an excellent starting point within the appropriate parameter space. The differences between the CNN-predicted and fully refined CeO_2_ lattice constants are of the order of 0.0001 Å, which is negligible.

The Tb_2_BaCoO_5_ results are presented in the second row of Fig. 3 ▸(*b*). The refinement starting from CNN-predicted values achieved an *R*_wp_ of 20.5%, which can be compared with 20.1% for the conventional manual refinement. This difference of only 0.4% emphasizes the network’s effectiveness in addressing the complexities inherent in this orthorhombic structure. Tb_2_BaCoO_5_ crystallizes in the space group *Immm* with nine crystallographically distinct atomic sites, presenting a more complex refinement problem than cubic CeO_2_. Extracted lattice parameters from the CNN and conventional Rietveld refinement are only within 0.06% difference.

The capability of our CNN model is further validated for the orthorhombic PbSO_4_ structure, as presented in Fig. 3 ▸(*c*). PbSO_4_ has a lower crystal symmetry and a more complex diffraction pattern, featuring numerous peaks concentrated within a narrow 2θ range. The orthorhombic structure necessitates the simultaneous determination of three independent lattice parameters: *a*, *b* and *c*. Despite the increased number of parameters, the CNN parameter predictions provided good structural and profile parameters. When initialized with these predicted values, the refinement converged to results that were virtually indistinguishable from those obtained through conventional methods. Specifically, the refinement starting from CNN-predicted values achieved an *R*_wp_ of 7.18%, differing from the traditional refinement result (7.16%) by only 0.02%, a negligible difference well below the statistical significance threshold. This performance indicates that the model can apply to various crystal structures. Our results suggest that the CNN has effectively learned to extract structural information from diffraction patterns and can provide sufficiently accurate parameters to determine the structure automatically. They can ensure reliable optimization in subsequent automatic or manual refinement procedures if needed. Additional refinements with ML for enhanced noise using CeO_2_ and PbSO_4_ systems (Appendix *G*[App appg]) and the quantification of uncertainty in the refined parameters from the CNN method for all three compounds further validate the robustness of our methodology (Appendix *H*[App apph]).

In summary, our automatic CNN method successfully produced high-quality Rietveld refinements for CeO_2_, Tb_2_BaCoO_5_ and PbSO_4_, which are consistent with the results obtained from conventional refinement within the bounds of experimental uncertainty. Consequently, the final refined structure obtained using CNN-predicted starting parameters is essentially indistinguishable from that acquired using traditional methods. This outcome is significant, as it suggests that the model can effectively replace the trial-and-error process of human initialization while maintaining accuracy.

### 4.2. Correlation analysis

**Figure 4:**
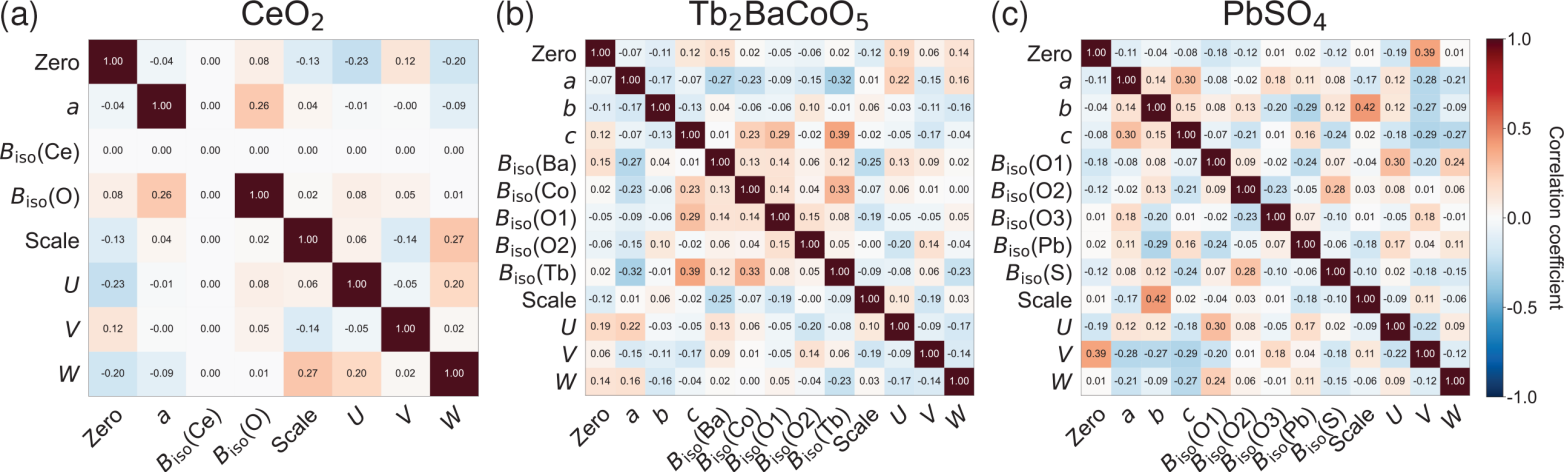
Correlation heatmaps for CNN models trained on XRD data from (*a*) CeO_2_, (*b*) Tb_2_BaCoO_5_ and (*c*) PbSO_4_. The matrices display Pearson correlation coefficients between Rietveld refinement parameters, with red indicating positive correlations and blue indicating negative correlations. The color intensity corresponds to the correlation strength, with darker colors representing stronger correlations.

To enhance our understanding of the relationships among the refinement parameters obtained through the CNN method and to validate the automatic refinement, we conducted a Pearson correlation analysis (Pearson, 1896 ▸). Fig. 4 ▸ presents the obtained correlation heatmaps of the parameters for all three crystal structures. These heatmaps reveal distinct patterns of parameter interdependencies that reflect the underlying crystallographic constraints and instrumental effects (McCusker *et al.*, 1999 ▸). The correlation matrix presented in Fig. 4 ▸(*a*) is straightforward. Specifically, the strongest correlation identified, with a value of 0.27, occurs between the scale factor and the peak-shape parameter *W*. Lattice parameter *a* exhibits a positive correlation with *B*_iso_(O) at +0.26. The zero-shift parameter displays negative correlations with the profile parameters *U* at −0.23 and *W* at −0.20. These weak to moderate correlations are consistent with the previous report on CeO_2_ that revealed no significant correlations among parameters obtained in the conventional Rietveld refinement (Balzar *et al.*, 2004 ▸). The observed zero correlation concerning *B*_iso_(Ce) arises because this parameter was fixed.

The Tb_2_BaCoO_5_ correlation matrix presented in Fig. 4 ▸(*b*) reveals more complex relationships among the parameters due to its lower-symmetry structure. The strongest positive correlation, measured at 0.39, occurs between the lattice parameter *c* and *B*_iso_(Tb). On the basis of the reported Tb_2_BaCoO_5_ structural analysis (Upadhyay & Sampathkumaran, 2018 ▸), this correlation suggests that *c*-axis expansion provides Tb atoms, located between CoO_2_ layers, with additional space for thermal vibration, resulting in higher *B*_iso_(Tb) values. Conversely, *c*-axis contraction restricts their motion. In contrast, the strongest negative correlation of −0.32 between the lattice parameter *a* and *B*_iso_(Tb) indicates competing effects along different crystallographic directions. Notable correlations also exist between *B*_iso_(Co) and the lattice parameter *c*, with a value of 0.23, as well as between *B*_iso_(Co) and *B*_iso_(Tb) at 0.33. These findings reflect the degrees of correlation between parameters in a given crystal structure and refinement.

Fig. 4 ▸(*c*) shows the correlation heatmap of PbSO_4_. The strongest positive correlation of 0.42 is observed between the lattice parameter *b* and the scale factor, indicating that variations along the *b* axis significantly affect diffraction intensity. This moderate correlation value aligns with previous orthorhombic refinements, which reported maximum correlations of 0.48 (Groen *et al.*, 1987 ▸), and remains well below problematic thresholds that could compromise refinement stability (Hill, 1992 ▸). The profile parameters exhibit expected coupling, with the zero shift and the peak-shape parameter *V* displaying a correlation of 0.39. Among the structural parameters, the lattice parameter *c* shows a negative correlation of −0.24 with *B*_iso_(S). This negative correlation is attributed to the constrained motion of the SO_4_ tetrahedron. Previous structural analysis of barite-group sulfates suggests that the rigid SO_4_ unit (Jacobsen *et al.*, 1998 ▸) undergoes restricted movement due to close packing along the *c* axis, which elucidates why *B*_iso_(S) decreases with *c*-axis contraction. The correlation patterns reveal how the CNN learns relationships between parameters during training. Strong correlations correspond to physically coupled parameters, such as lattice constants and thermal parameters, whereas weak correlations mark parameters that can be refined independently (see Appendix *I*[App appi]).

## 5. Discussion

Building on the validation metrics presented above, our CNN approach demonstrates robust performance across crystal structures of varying complexity and powder XRD data. This is further supported by successful ML refinement using a complex monoclinic structure (see Appendix *J*[App appj]). Together, the studies show that CNNs trained on simulated powder patterns can function as reliable automated refinement engines. By combining neural network parameter estimates with conventional Rietveld least squares, we attain crystallographic precision comparable to manual refinement (McCusker *et al.*, 1999 ▸). The present results highlight the considerable promise of CNN-driven large-scale powder diffraction analysis.

Future research can readily expand this framework to encompass the complete range of crystal systems, including triclinic structures, as well as multiphase mixtures. This approach can be easily applied to powder XRD data on many other inorganic, as well as organic, materials. Moreover, this CNN methodology can be applied to other types of diffraction data, such as powder neutron diffraction, as they share the same working principles. Furthermore, this method can be integrated with high-throughput diffraction experiments, and real-time analysis pipelines could enable autonomous materials characterization at the scale and speed required for rapid materials discovery and characterization (Szymanski *et al.*, 2021 ▸, 2023 ▸). This approach establishes a foundation for fully automated powder diffraction analysis while maintaining the crystallographic rigor essential for scientific research by providing consistent and accurate parameter determination with minimal manual intervention.

## 6. Conclusions

This study demonstrates that a CNN-based algorithm can reliably perform automatic Rietveld refinements, yielding results comparable to those obtained from conventional manual refinement within the bounds of experimental uncertainties. We propose an automatic Rietveld refinement methodology applicable to various materials. This study highlights the potential of deep learning methods for a wide range of applications in both fundamental research and industrial settings.

## Figures and Tables

**Figure 5:**
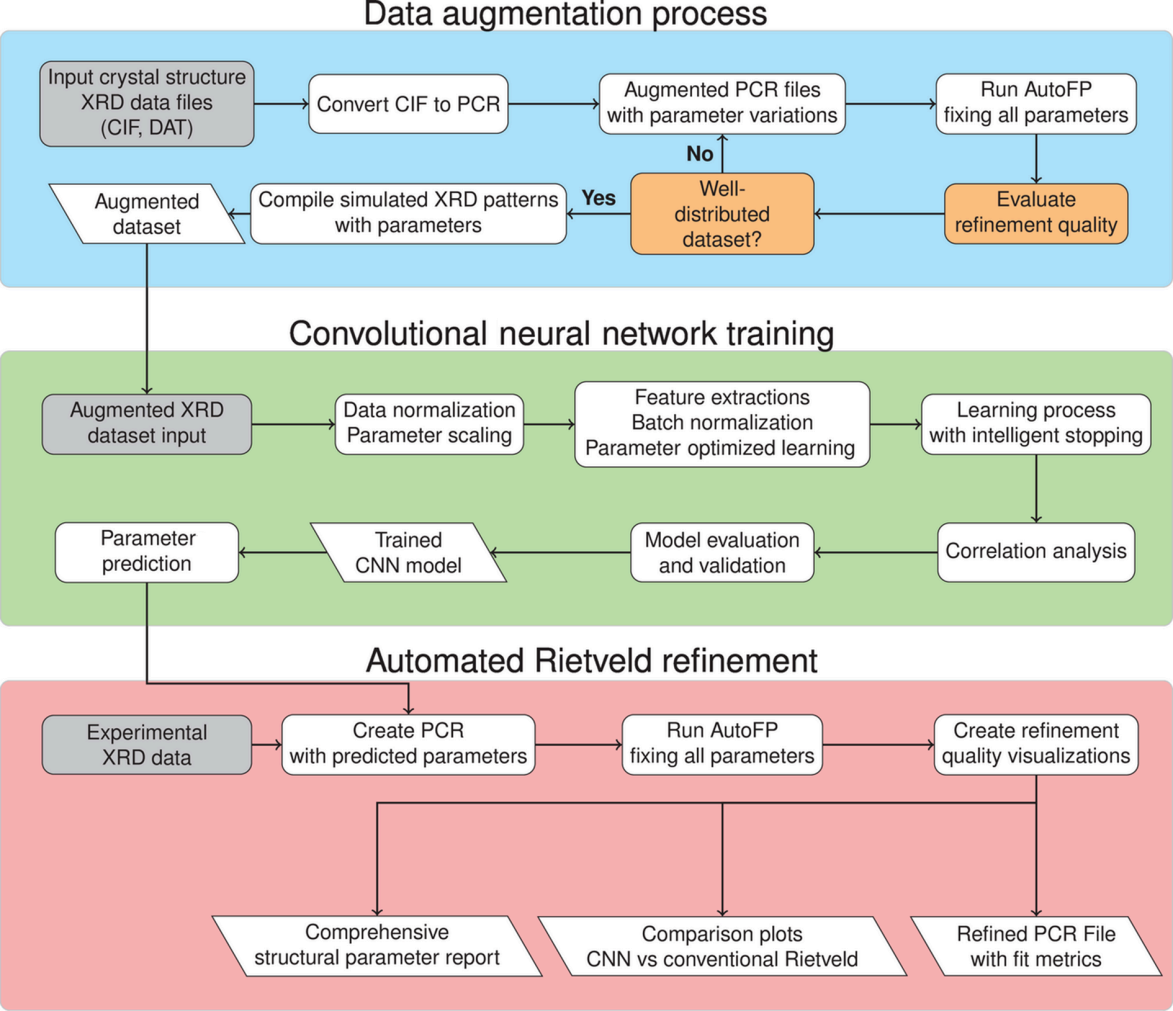
XRD analysis pipeline with three major sections. The data-augmentation process transforms crystal structure files and XRD data into a refinement format with systematic parameter variations. These undergo structural refinement and quality evaluation through an adaptive feedback loop to produce a comprehensive training dataset. The CNN training section applies feature extraction, batch normalization and parameter-optimized learning paths to this dataset. The resulting model includes correlation analysis of parameter relationships. The automated Rietveld refinement section applies this model to real-world diffraction patterns. It creates refinement files with predicted parameters, conducts automated structure refinement and produces visualizations that compare the CNN results with those from traditional methods.

**Figure 6:**
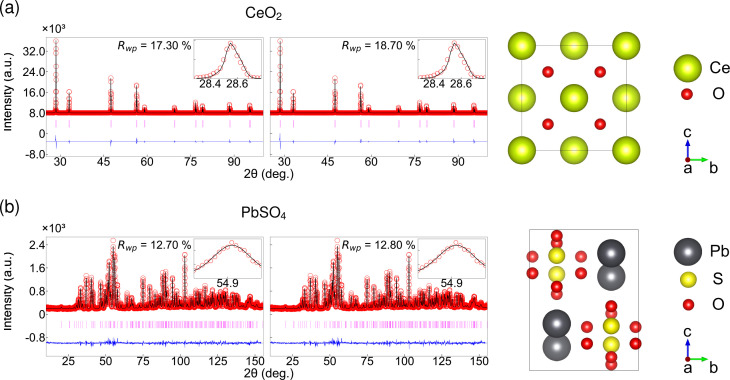
Noise robustness validation for (*a*) CeO_2_ and (*b*) PbSO_4_ with 10% added noise. The left and middle panels display conventional and CNN ML refinements, respectively. Each plot displays observed data (red circles), calculated patterns (black lines), difference curves (lower blue traces) and Bragg positions (magenta markers). The close agreement between CNN and conventional refinement *R*_wp_ values demonstrates robust performance under noisy conditions. The right panels show the refined crystal structure from the ML method.

**Figure 7:**
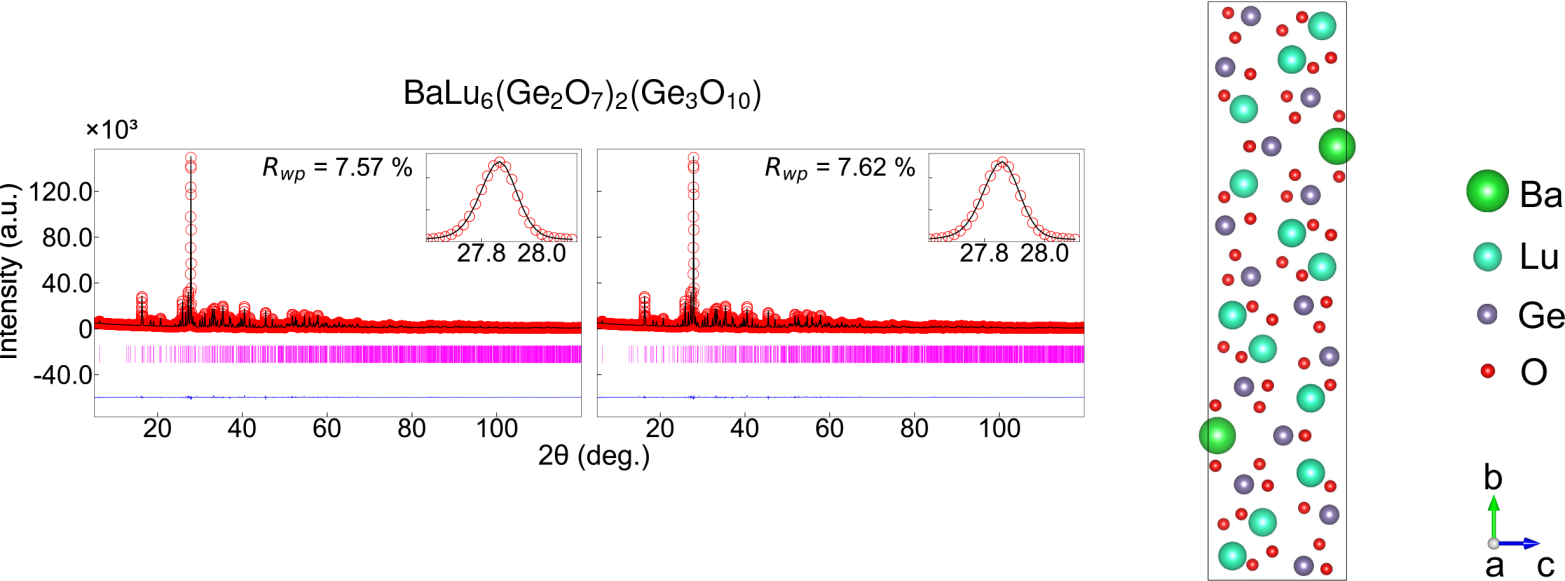
Rietveld refinement results for BaLu_6_(Ge_2_O_7_)_2_(Ge_3_O_10_) with a monoclinic structure *P*2_1_/*m*. The left panel displays conventional Rietveld refinement results (*R*_wp_ = 7.57%); the middle panel shows CNN-predicted refinement (*R*_wp_ = 7.62%). Each plot shows observed data as red circles, calculated patterns as black lines, difference curves (lower blue traces) and Bragg positions as magenta markers. Insets provide zoomed views of the strongest peak. The crystal structure with coordinate axes (*a*, *b*, *c*) is shown on the right.

**Figure 8:**
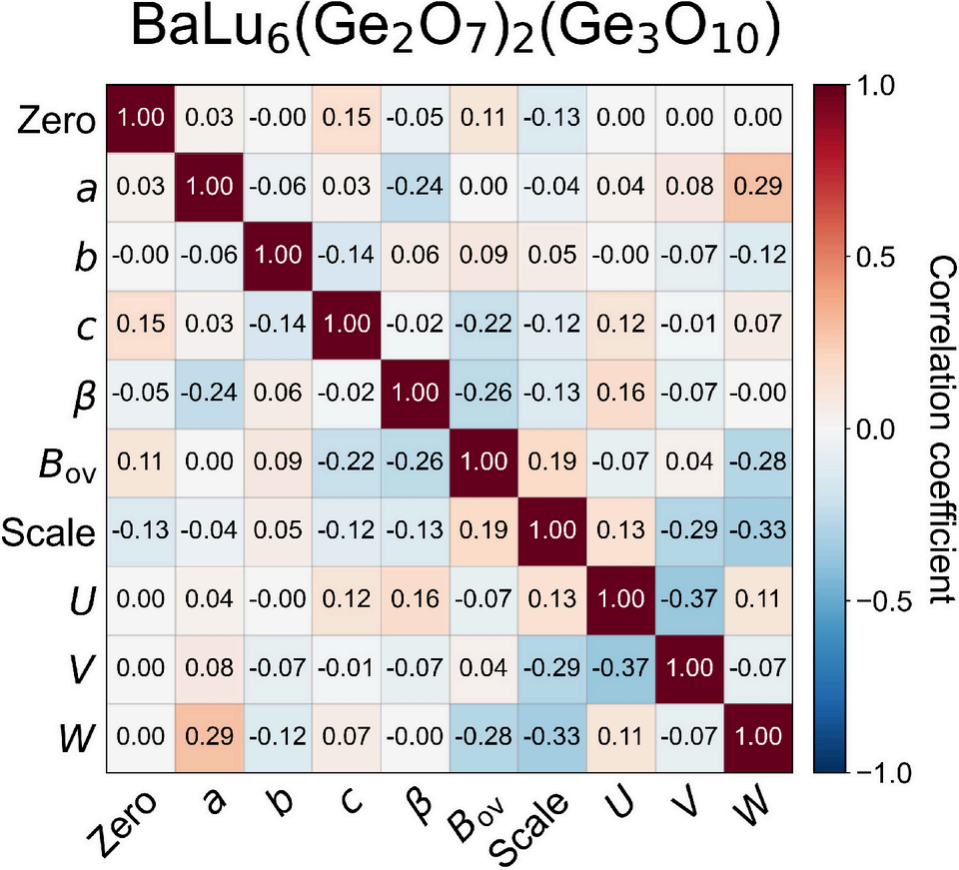
Parameter correlation matrix for BaLu_6_(Ge_2_O_7_)_2_(Ge_3_O_10_) CNN prediction. The heatmap shows Pearson correlation coefficients between all ten predicted parameters. Color scale ranges from −1 (negatively correlated) to +1 (positively correlated). The relatively weak correlations (mean |*r*| = 0.11) indicate that parameters are largely independent.

**Table 1:** Comparison of parameters obtained from the CNN predictions and conventional Rietveld refinement for CeO_2_, Tb_2_BaCoO_5_ and PbSO_4_ The *R* factors for *R*_p_ and *R*_wp_ are in percent, zero shift is in degrees, the lattice constants (*a*, *b*, *c*) are in ångstrom, the isotropic displacement factors (*B*_iso_) are in Å^2^, and scale is arbitrary, whereas the peak-shape coefficients *U*, *V* and *W* are dimensionless. Standard deviations are given for the Rietveld refinements in parentheses.

	CeO_2_		Tb_2_BaCoO_5_		PbSO_4_	
	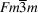		*Immm*		*Pnma*	
Parameter	Rietveld	CNN	Rietveld	CNN	Rietveld	CNN
*R*_p_	12.6	12.7	17.2	17.6	7.37	7.38
*R*_wp_	16.7	17.3	20.1	20.5	7.16	7.18

Zero shift	−0.0185 (11)	−0.018885	−0.0208 (11)	−0.020920	−0.0880 (33)	−0.089054
*a*	5.41143 (4)	5.411551	3.75597 (7)	3.755761	8.47884 (15)	8.478931
*b*	–	–	5.82381 (11)	5.823482	5.39673 (10)	5.396857
*c*	–	–	11.55177 (21)	11.550838	6.95840 (13)	6.958322

*B*_iso_(Ce)	0.040 (47)	0.039679	–	–	–	–
*B*_iso_(O)	0.849 (266)	0.855267	–	–	–	–
*B*_iso_(Ba)	–	–	0.030 (28)	0.030453	–	–
*B*_iso_(Tb)	–	–	0.030 (28)	0.030454	–	–
*B*_iso_(Co)	–	–	0.030 (28)	0.030453	–	–
*B*_iso_(O1)	–	–	0.030 (28)	0.030453	1.987 (35)	1.987669
*B*_iso_(O2)	–	–	0.030 (28)	0.030454	1.471 (33)	1.467085
*B*_iso_(O3)	–	–	–	–	1.321 (24)	1.317186
*B*_iso_(Pb)	–	–	–	–	1.416 (24)	1.411911 ▸
*B*_iso_(S)	–	–	–	–	0.420 (44)	0.418023

Scale	0.000644 (6)	0.000667	0.000434 (2)	0.000433	1.4807 (52)	1.480242
*U*	0.0216 (24)	0.021726	0.0269 (23)	0.026863	0.1556 (33)	0.154734
*V*	−0.0397 (30)	−0.039948	−0.0275 (26)	−0.027412	−0.4559 (74)	−0.453976
*W*	0.0197 (9)	0.019823	0.0167 (8)	0.016649	0.4191 (45)	0.417253

**Table 2:** Comprehensive evaluation metrics for CNN versus conventional Rietveld refinement

Material	Method	*R*_wp_	*R*_p_	*R*_exp_	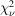
CeO_2_	Rietveld	16.70	12.60	4.10	16.47
	CNN	17.30	12.70	4.12	17.56

PbSO_4_	Rietveld	7.16	7.37	3.54	4.09
	CNN	7.18	7.38	3.54	4.11

Tb_2_BaCoO_5_	Rietveld	20.10	17.20	7.39	7.39
	CNN	20.50	17.60	7.39	7.71

BaLu_6_(Ge_2_O_7_)_2_(Ge_3_O_10_)	Rietveld	7.57	6.11	2.90	6.80
	CNN	7.62	6.17	2.90	6.90

**Table 3:** MC dropout uncertainty estimates for all predicted parameters Uncertainties represent one standard deviation.

Material	Parameter	Mean (*T* = 100)†	Std (*T* = 100)†
CeO_2_	Zero	−0.018922	0.000297
	*a* (Å)	5.396889	0.037379
	*B*_iso_(Ce)	0.039656	0.000388
	*B*_iso_(O)	0.854779	0.008364
	Scale	0.000668	0.000014
	*U*	0.022034	0.000516
	*V*	−0.040526	0.000959
	*W*	0.020103	0.000469

PbSO_4_	Zero	−0.088883	0.000464
	*a* (Å)	8.478525	0.006225
	*b* (Å)	5.396629	0.003951
	*c* (Å)	6.958030	0.005099
	*B*_iso_(O1)	1.986855	0.001660
	*B*_iso_(O2)	1.466467	0.001244
	*B*_iso_(O3)	1.316636	0.001105
	*B*_iso_(Pb)	1.411334	0.001166
	*B*_iso_(S)	0.417859	0.000338
	Scale	1.478696	0.001788
	*U*	0.154858	0.000575
	*V*	−0.454345	0.001726
	*W*	0.417591	0.001582

Tb_2_BaCoO_5_	Zero	−0.020490	0.000376
	*a* (Å)	3.733601	0.001318
	*b* (Å)	5.788520	0.002077
	*c* (Å)	11.483374	0.003655
	*B*_iso_(Ba)	0.030303	0.000018
	*B*_iso_(Co)	0.030303	0.000018
	*B*_iso_(O1)	0.030304	0.000018
	*B*_iso_(O2)	0.030303	0.000018
	*B*_iso_(Tb)	0.030303	0.000018
	Scale	0.000432	<0.000001
	*U*	0.026802	0.000079
	*V*	−0.027349	0.000082
	*W*	0.016613	0.000046

† Computed from *T* = 100 stochastic forward passes through the CNN with dropout enabled (*p* = 0.2).

## Data Availability

Methods and data discussed in this work are publicly available at https://github.com/DataForgeSci/RAPID. This contains the code of the Python implementaion of RAPID, installation manual, program manual and table of contents.

## Appendix A Computational performance

All computations were performed on a workstation equipped with an AMD Ryzen 9 9950X 16-core processor, 126 GB RAM, and a NVIDIA GeForce RTX 5070 Ti GPU with 16 GB VRAM. For training datasets of 10 000 synthetic diffraction patterns, CNN model training converges within 100 epochs in ∼6 min on the GPU. The measured training rate is ∼8 s per epoch for 10 000 training patterns including validation.

Once a trained model is obtained, inference on experimental diffraction patterns is near-instantaneous. A single CNN forward pass requires ∼2 ms per pattern. For MC dropout (see Appendix *H*[App apph]) uncertainty estimation with *T* = 100 stochastic forward passes, the total inference time is ∼240 ms per pattern. The peak GPU memory usage is ∼12 MB. This represents a substantial speedup compared with traditional Rietveld refinement, which typically requires 10–30 min of manual parameter adjustment per sample (McCusker *et al.*, 1999 ▸). Iterative optimization approaches such as black-box optimization (Ozaki *et al.*, 2020 ▸) require ∼1 h per sample for 200 optimization trials.

All computational operations scale linearly *O*(*n*) with the number of training samples or diffraction data points. These operations include data generation via *FullProf* simulation, CNN training and inference. The one-time training cost is greatly amortized across numerous subsequent predictions.

## Appendix B Prior phase identification

Accurate determination of the crystalline phase in a powder diffraction pattern is a prerequisite for subsequent Rietveld refinements. In conventional Rietveld refinement, an unknown diffraction pattern is compared with reference databases to identify candidate phases. Recent studies have automated phase prediction for complex multiphase systems (Lee *et al.*, 2020 ▸; Szymanski *et al.*, 2021 ▸) using ML approaches, ranked by similarity scores (Mikhalychev & Ulyanenkov, 2017 ▸).

In this work, we assume that the phase has been predetermined. A user identifies the crystalline phase using conventional methods, such as matching with a database. It can be also done using automatic methods, such as the ML classifiers developed by Lee *et al.* (2020 ▸) and autonomous phase-mapping approaches (Szymanski *et al.*, 2021 ▸). Consequently, phase identification is not performed by the neural network pipeline presented here. This indicates that any residual error in subsequent structural refinements arises from the parameter prediction task rather than the hidden phase. This study exclusively focuses on structural refinement and profile parameters for the assigned phase. After obtaining the corresponding CIF with the structural model, the trained CNN can be used for automated parameter refinement. This separation of phase identification from parameter refinement allows for the independent optimization of each component, facilitating integration with existing laboratory workflows.

Our pipeline could potentially be integrated with automated phase-identification software (Lee *et al.*, 2020 ▸; Szymanski *et al.*, 2021 ▸; Mikhalychev & Ulyanenkov, 2017 ▸). A modular workflow could be developed for future projects. The experimental powder XRD data can be used for phase identification, CIF retrieval and automatic Rietveld refinement. The most probable structural candidate from the phase-identification module can be selected. The information on the identified phase, including the compound name and space group, can be used to obtain the corresponding CIF from the database utilized by the phase-determination programs (Lee *et al.*, 2020 ▸; Szymanski *et al.*, 2021 ▸; Mikhalychev & Ulyanenkov, 2017 ▸). This CIF serves as the entry point to our RAPID pipeline, outputting refined structural parameters with reliability metrics, including *R*_wp_, as a fitting quality indicator. Such integration would enable end-to-end automation from an unknown experimental diffraction pattern to refined structural parameters. Implementing and developing this integrated workflow in a single program and extending it to multiphase structural analysis represents important future work.

## Appendix C Background treatment

Background parameters are usually among the first to be estimated in the Rietveld refinement sequence to avoid instabilities that may arise when correlated parameters are refined simultaneously (Toby, 2024 ▸). It is common practice to keep these parameters fixed during subsequent structural refinements to reduce parameter correlations and improve stability (Kisi & Howard, 2008 ▸). A practical approach for establishing these initial parameters is the Le Bail extraction, which is a whole-pattern decomposition method that refines unit cell, profile and background parameters while allowing all reflection intensities to vary freely instead of being constrained by a structural model. This method provides reliable starting values for subsequent Rietveld refinements, during which atomic positions and occupancies constrain intensities. For stable fitting, background parameters obtained from Le Bail extraction are typically held constant and only relaxed after achieving a satisfactory fit (Kisi & Howard, 2008 ▸). Following this practice, the background coefficients in this study were fixed during data generation, CNN training and validation, with the option to refine them once the fit was satisfactory. This stepwise strategy is consistent with the International Union of Crystallography guidelines for reliable Rietveld refinements (McCusker *et al.*, 1999 ▸).

## Appendix D Data augmentation

The *AutoFP* program generates and processes input data files. It controls the widely utilized refinement program *FullProf*. The initial data preparation comprises three main components: the CIF, an experimental X-ray file (DAT) and a control file (PCR), as an input file for the refinement.

The data-augmentation pipeline is illustrated in Fig. 5 ▸. The CIF to PCR conversion process extracts crystallographic information and formats it according to the *FullProf* file template (Rodríguez-Carvajal, 1993 ▸). The generation of parameter variations produces multiple PCR files with systematically modified parameters. The system varies eight categories of parameters within the bounds specified in the input file. The zero shift and background coefficients accept absolute variation ranges, whereas the lattice parameters, isotropic displacement parameters, scale factor and peak-shape parameters (*U*, *V* and *W*) utilize percentage-based variations. The implementation supports variations of *B*_iso_ for each atom, allowing for distinct displacement parameter adjustments for each unique atom in the structure. Displacement parameters for each atom are fitted individually.

Parameter sampling utilizes two complementary strategies: grid points for perfect cube numbers and Sobol sequences for quasirandom uniform sampling. Random sampling generates additional parameter combinations by utilizing uniform random distributions within specified bounds. The total number of datasets is equally divided between the uniform and random sampling methods. Dataset generation can range from hundreds to tens of thousands, typically involving 10 000 datasets for comprehensive exploration of the parameter space and optimal performance when training the subsequent CNN model.

A critical component of the pipeline is quality evaluation, in which we categorize the refinement results into three classes (Close, Boundary and Invalid) according to the weighted profile *R* factor (*R*_wp_):  for Close,  for Boundary and otherwise Invalid. The reference value *R*_wp_(ref) is obtained from the Rietveld refinement of the first synthetic sample, which uses the original CIF parameters, and the system features an adaptive mechanism that automatically adjusts parameter ranges to achieve ∼80% Close and 20% Boundary distributions.

## Appendix E CNN algorithm and training optimization

This section provides comprehensive details regarding the CNN algorithm. The transformation from raw XRD data to predicted structural and profile parameters consists of two main components. The first component is a convolutional feature extraction module that processes the input XRD intensity data. The second component is a fully connected regressor that maps the extracted features to crystallographic parameters. This architecture encapsulates the end-to-end learning process, in which the network predicts physical parameters from diffraction patterns without manual intervention.

Fig. 5 ▸ illustrates the CNN pipeline, comprising three main components: the data-augmentation process, CNN training and automated Rietveld refinement. The simulated powder XRD patterns, along with the corresponding structural and profile parameters, are incorporated into the CNN architecture as a one-dimensional array known as the kernel. The CNN processes these inputs through a sequence of convolutional and pooling layers. This process yields refined predictions for physical parameters, including the zero shift, background coefficients, lattice parameters, *B*_iso_, scale factor and peak-shape parameters (*U*, *V* and *W*).

The input XRD data undergo normalization and centering before processing. Each intensity pattern is divided by its maximum value, and its mean is subtracted to standardize the input range. This preprocessing step ensures consistent learning, irrespective of the absolute intensity scales present in the simulated data. This is done for both simulated and experimental data.

To ensure completeness, we provide the CNN convolution details omitted in Fig. 2 ▸ for brevity. The second and third blocks utilize kernel sizes of 5 with a padding of 2 to emphasize localized features. The second block increases the channel count to 128 while maintaining the same operational sequence: convolution, ReLU activation, batch normalization and pooling. The third block maintains 128 channels while further reducing spatial dimensions. The fourth and fifth blocks employ smaller kernel sizes of 3 with a padding of 1. The fourth block increases the channels to 256 to enhance specialized feature detection, while the fifth block reduces them back to 128. The adaptive pooling layer guarantees a fixed output size of 4, regardless of input length. This results in a feature shape of [batch size, 128, 4].

Each structural or profile parameter is considered within its corresponding output head in the CNN code. The zero-shift output head comprises three fully connected layers (64–32–1) that predict the zero offset with high precision, as this parameter significantly affects peak positions. The background output head uses two fully connected layers (32–1) to predict the background coefficient. By default, this background output head is trained; however, it can be excluded when analyzing materials or experimental setups with negligible or preprocessed background contributions. The *B*_iso_ output head (64–32–) predicts  displacement factors, where  corresponds to the number of unique atom types in the structure. This enables accurate modeling of thermal motion in complex materials. The scale-factor output head (64–32–1) predicts the global intensity scaling factor, which influences the overall amplitude of the pattern. The peak-shape output head (64–3) jointly predicts three peak-shape parameters, capturing their inherent correlations for accurate modeling of peak profiles. This architecture offers several advantages. Parameters of varying magnitudes and physical significance receive appropriate scaling and specialized treatment. Crystal symmetry constraints are naturally incorporated through variable output lattice parameters. Each output head is independently optimized with target-specific loss weighting.

For the cross-checking of the training results, we divided the available data into a training set and a test set. This split ensures that the network has enough data to learn patterns during the training phase while retaining a still meaningful portion for validation.

For training, we used the Adam optimizer (Kingma & Ba, 2017 ▸) with smooth L1 loss (Terven *et al.*, 2025 ▸; Girshick, 2015 ▸), a learning rate of 10^−3^ and a weight decay of 10^−4^ for L2 regularization (Loshchilov & Hutter, 2019 ▸). Training was conducted with a batch size of 128 and parameters were initialized using LeCun initialization (LeCun *et al.*, 1998*b* ▸). We employed a ReduceLROnPlateau scheduler (PyTorch Con­tributors, 2024 ▸) that monitors validation loss and reduces the learning rate by a factor of 0.5 when improvements plateau, with a patience of 5 epochs. Early stopping was implemented with a patience of 7 epochs, terminating training when target-specific error thresholds were achieved or the maximum of 100 epochs was reached.

In addition to parameter scaling, we apply target-specific weights (*w*_*i*_) to address the remaining scale disparity. A lower loss weight is applied to the zero-shift offset than to the lattice or profile terms. Typical zero-shift errors are smaller than lattice constants or profile widths in their native units (Das *et al.*, 2025 ▸; Ermrich & Opper, 2013 ▸). Without reweighting, the zero-shift gradient would be much smaller than those of the larger-scale parameters, which would effectively silence it. This weighting ensures that each output generates gradients of comparable magnitude during backpropagation, preventing large-scale terms from dominating the optimization (Vizoso & Dingreville, 2025 ▸; Röcken & Zavadlav, 2024 ▸). The weighting strategy is also guided by downstream sensitivity during Rietveld refinement. Zero shifts within the refinement capture range can be easily corrected, while inaccurate scale or *B*_iso_ values can trap the refinement in local minima.

We implemented a target-specific early stopping mechanism that recognizes varying training rates across crystallographic parameters (Caruana, 1997 ▸). This strategy establishes distinct error thresholds based on the significance of the crystallographic parameters. The lattice constants require the highest precision. The scale factors also necessitate high accuracy. The *B*_iso_ and zero-shift parameters allow slightly larger errors. Training continues until the validation loss stabilizes. The system ensures all target-specific thresholds are met before termination. If critical parameters fail to meet their precision requirements after the standard patience period, training is automatically extended with a reduced learning rate. This differential precision approach reflects the varying importance of each crystallographic parameter in achieving successful Rietveld refinement.

Incorporating a larger fraction of real experimental data is challenging because our approach trains from a single structural file for each compound, unlike previous methods that use large databases containing many different compounds. Domain adaptation or fine-tuning with real experimental data represents an important direction for future development.

## Appendix F Evaluation metrics

To assess refinement quality, we report standard crystallographic metrics following the *FullProf* manual: the weighted profile *R* factor (*R*_wp_), the unweighted profile *R* factor (*R*_p_), the expected *R* factor (*R*_exp_) and the reduced chi-squared (). Table 2 ▸ presents these metrics for all validated materials.

The close agreement in *R*_p_ and  between CNN and conventional refinement demonstrates that the CNN predictions achieve comparable pattern-matching quality across all crystal systems tested.

The CNN directly predicts the Caglioti peak-width parameters (*U*, *V*, *W*) to ensure correct peak shapes. Visual inspection of difference curves confirms the absence of systematic peak-shape misfits.

Iterative least-squares refinement can converge to different local minima depending on starting parameters. In contrast, the CNN performs direct prediction in a single forward pass (Sulam *et al.*, 2020 ▸; Papyan *et al.*, 2017 ▸). The output is deterministic and the same input pattern always yields the same predicted parameters. This eliminates sensitivity to initial conditions.

## Appendix G Robustness validation with enhanced noise

To assess robustness, CNN models were trained on datasets with added noise. The noisy intensity *I*_noisy_ is generated as

where η is the noise level (0.1 for 10% noise) and *U*(−1, 1) is a uniform random variable. Fig. 6 ▸ presents results for 10% noise. For CeO_2_, the CNN achieved *R*_wp_ = 18.7% while the con­ven­tional *R*_wp_ = 17.3%, with a difference of 1.4%. For PbSO_4_, the CNN achieved *R*_wp_ = 12.8% while the conventional *R*_wp_ = 12.7%, with a difference of only 0.1%. The small differences confirm robustness to typical experimental noise levels.

The simulated diffraction data encompass a wide range of experimental situations, including extrinsic effects from instrument drift (Cline *et al.*, 2015 ▸). Variation in instrumental characteristics affects peak shapes and positions, including zero shifts. To mitigate these extrinsic effects, the physics-informed simulation perturbs the peak-broadening parameters *U*, *V* and *W* (Caglioti *et al.*, 1958 ▸), as well as asymmetry parameters and the zero-degree offset during simulated data generation. The peak-broadening parameters govern the variation of diffraction peak widths with scattering angle, while the zero-degree offset accounts for systematic shifts in the 2θ scale. By sampling these parameter ranges during training, the CNN learns to accommodate various instrumental conditions.

## Appendix H Uncertainty quantification

Standard deep learning tools for regression do not inherently capture model uncertainty. To provide probabilistic outputs and increase the trustworthiness of results, we employ Monte Carlo (MC) dropout (Gal & Ghahramani, 2016 ▸).

MC dropout provides uncertainty estimates by performing *T* stochastic forward passes with dropout enabled during inference. For each parameter *j*, the predictive mean μ_*j*_ and uncertainty σ_*j*_ are computed as

where  is the prediction from the *t*th forward pass. Table 3 ▸ presents MC dropout uncertainties for the three validated materials. The small standard deviations observed for most parameters indicate that the CNN predictions are stable and consistent across multiple forward passes. Values reported as <10^−6^ indicate standard deviations below the numerical precision of the table.

The MC dropout uncertainties are comparable to or smaller than the corresponding uncertainties from conventional Rietveld fitting (Table 1 ▸) for most parameters. This demonstrates that the CNN provides stable predictions for these parameters.

## Appendix I Correlation analysis

This section presents a detailed methodology for the correlation analysis of refinement parameters in automatic ML refinement. To achieve this, we adopt an approach that mirrors the processes employed by conventional Rietveld algorithms to extract correlations between parameters.

In traditional refinement, the algorithm explores the parameter space to identify multiple plausible parameter sets that can explain the observed pattern (McCusker *et al.*, 1999 ▸). Similarly, we selected synthetic parameter sets satisfying the Close criterion (see Appendix *D*[App appd]). We followed established practices in which poor-quality refinements with high *R* factors are routinely excluded (Corriero *et al.*, 2023 ▸; Tsubota & Kitagawa, 2017 ▸). From our 10 000 synthetic XRD patterns, we selected 50 representative samples that met this criterion to compute the correlation matrix. By simulating the multiple plausible solutions that conventional Rietveld algorithms would extract from a single CIF, this approach permits meaningful correlation analysis. Evidence from previous studies demonstrates that well-fitted synthetic datasets replicate similar parameter correlations and uncertainties to experimental refinements (Tian & Billinge, 2011 ▸).

We computed the correlation matrix from the filtered parameter values for each of the three crystal structures: CeO_2_, Tb_2_BaCoO_5_ and PbSO_4_. The Pearson correlation coefficient (Saccenti *et al.*, 2020 ▸) measures the relationship between two parameters by comparing how each parameter deviates from its mean value. The correlation coefficients range from −1 to +1, where values close to +1 indicate a strong positive correlation, values close to −1 signify a strong negative correlation and values close to 0 suggest no linear relationship between the parameters. The correlation patterns observed in our CNN-derived matrices align with those reported in conventional Rietveld studies (Pani *et al.*, 1995 ▸; Groen *et al.*, 1987 ▸). Our results yield comparable values within the range typically reported for reliable single-phase refinements. Fig. 4 ▸ presents the correlation matrices for the three crystal structures. The observed correlation of *B*_iso_(Ce) with all other parameters is zero [Fig. 4 ▸(*a*)] as the variation was smaller than the numerical precision. We presented the correlation as zero for visualization purposes. This limitation does not affect the CNN’s ability to refine other parameters, as *B*_iso_(Ce) effectively becomes a fixed parameter in this case (Ballirano *et al.*, 2021 ▸).

## Appendix J Low-symmetry validation

To address concerns regarding validation on diverse crystal systems, we extended CNN-based parameter prediction to a monoclinic structure, which has lower symmetry than the cubic and orthorhombic systems validated in the main text. The monoclinic crystal system requires refinement of a β angle in addition to three independent lattice parameters (*a*, *b*, *c*). This provides a more stringent test of the methodology.

We selected BaLu_6_(Ge_2_O_7_)_2_(Ge_3_O_10_) (space group *P*2_1_/*m*, monoclinic) as a validation case for low-symmetry structures. The CIF was obtained from the Crystallography Open Database (Gražulis *et al.*, 2009 ▸) (COD entry 7713053; Lipina *et al.*, 2023 ▸) and the experimental powder XRD pattern was taken from the Open Experimental Powder X-ray Diffraction Database (opXRD) (Hollarek *et al.*, 2025 ▸).

The CNN achieved *R*_wp_ = 7.62% and conventional Rietveld refinement *R*_wp_ = 7.57%, a difference of only 0.05% (Fig. 7 ▸). This close agreement demonstrates that the CNN methodology successfully extends to lower-symmetry crystal systems with minimal loss in accuracy. The CNN-predicted monoclinic lattice parameters (*a*, *b*, *c*, β) and displacement parameter (*B*_ov_) closely match the conventional refinement values.

Fig. 8 ▸ presents the parameter correlation matrix for the monoclinic BaLu_6_(Ge_2_O_7_)_2_(Ge_3_O_10_) CNN prediction. The strongest positive correlation is 0.29 between the lattice parameter *a* and the peak-profile parameter *W*, while the strongest negative correlation is −0.37 between the peak-profile parameters *U* and *V*. The mean absolute correlation coefficient is 0.11.

The relatively weak inter-parameter correlations indicate that the ten predicted parameters are largely independent. The *U*–*V* negative correlation is consistent with the mathematical relationship between these peak-profile parameters in the Caglioti formula (Caglioti *et al.*, 1958 ▸).

The current implementation addresses single-phase refinement where the crystalline phase is known. Extension to multiphase systems, where multiple crystalline phases coexist in the same sample, represents an important direction for future work. This would require training on simulated multiphase patterns with varying phase fractions and developing architectures capable of predicting parameters for multiple phases simultaneously.
